# Influence of Diameter and Cyclic Mechanical Stimulation on the Beating Frequency of Myocardial Cell-Laden Fibers

**DOI:** 10.3390/gels9090677

**Published:** 2023-08-23

**Authors:** Stavroula Kyriakou, Andreas Lubig, Cilia A. Sandhoff, Yasmin Kuhn, Stefan Jockenhoevel

**Affiliations:** 1 Department of Biohybrid & Medical Textiles (BioTex), AME-Institute of Applied Medical Engineering, Helmholtz Institute, RWTH Aachen University, 52074 Aachen, Germany; kyriakou@ame.rwth-aachen.de (S.K.); sandhoff@ame.rwth-aachen.de (C.A.S.); kuhn@ame.rwth-aachen.de (Y.K.); 2 AMIBM-Aachen-Maastricht-Institute for Biobased Materials, Maastricht University, 186260 Geleen, The Netherlands

**Keywords:** fibrin gel, iPSC-CMs, biofabrication, mechanical stimulation, atrioventricular block

## Abstract

Atrioventricular block (AVB) is a severe disease for pediatric patients. The repetitive operations needed in the case of the pacemaker implantation to maintain the electrical signal at the atrioventricular node (AVN) affect the patient’s life quality. In this study, we present a method of biofabrication of multi-cell-laden cylindrical fibrin-based fibers that can restore the electrical signal at the AVN. We used human umbilical vein smooth muscle cells (HUVSMCs), human umbilical vein endothelial cells (HUVECs) and induced pluripotent stem cell cardiomyocytes (iPSC-CMs) cultivated either statically or dynamically to mimic the native AVN. We investigated the influence of cell composition, construct diameter and cyclic stretch on the function of the fibrin hydrogels in vitro. Immunohistochemistry analyses showed the maturity of the iPSC-CMs in the constructs through the expression of sarcomeric alpha actinin (SAA) and electrical coupling through Connexin 43 (Cx43) signal. Simultaneously, the beating frequency of the fibrin hydrogels was higher and easy to maintain whereas the concentration of iPSC-CMs was higher compared with the other types of cylindrical constructs. In total, our study highlights that the combination of fibrin with the cell mixture and geometry is offering a feasible biofabrication method for tissue engineering approaches for the treatment of AVB.

## 1. Introduction

The atrioventricular block is a crucial pathological situation that specifically constitutes a significant burden in pediatric heart surgery. The atrioventricular block of any origin-immune-mediated, inherited, or apparently idiopathic- [1] is a rare [2] pathological situation that belongs to the huge category of bradycardias [3]. Cardioneuroablation [4], pacemaker implantation, and antibiotics have been used for the treatment of the atrioventricular block [5]. Nevertheless, pacemaker implantations necessitate repetitive operations and influence the life quality of the children [6,7]. Particularly, pacemaker implantation has been associated with left ventricular (LV) dysfunction [8], dilated cardiomyopathy [9,10] relatively high mortality rate in pediatric patients after the surgery [11], risk of syncope, heart failure and sudden death [12]. 

Many studies have reported solutions that could be potentially useful for the cell-mediated therapy of atrioventricular block. Gorabi et al. inserted a Tbx18 gene alone and also Tbx18-inserted pacemaker-like stem cells in a complete heart block rat model achieving a pacemaker capacity by comparing the outcomes of the gene and cell insertion [13]. Lu et al. injected mHCN4-modified cMSCs with support of an electronic pacemaker [14]. In another study, Yokokawa et al. inserted mesenchymal stem cells with antifibrotic action in a rat model of complete atrioventricular block [15]. Moreover, Chaveau et al. used iPSC-CMs as therapy for the atrioventricular block, but the beating frequency and other parameters related to pacing remain still unraveled [16]. To improve particular aims on cell orientation and achieve adequate perfusion parameters for cardiovascular applications many studies have developed bioreactors. Cingolani et al. created a strip of cardiomyocytes by attaching the cells to magnetic beads that were then aligned over a magnetic field [17]. Using a rat model of a complete heart block, they implanted this strip outside of the heart, restoring atrioventricular conduction. Miklas et al. [18] prepared collagen gels embedded with neonatal rat heart-derived cardiomyocytes performing electrical and mechanical stimulation. Additionally, Nunes et al. [19] created self-assembled electrically stimulated cardiac biowires using iPSC-CMs. Moreover, Kensah et al. [20] applied cyclic and static stretch in murine and human-induced cardiomyocytes. In terms of the geometry, Keijdener et al. [21] established a method for the preparation of cylindrical fibrin gels using neural Schwann cells, endothelial and smooth muscle cells showing orientation after applied stretch of different degrees. 

Nowadays the treatment of atrioventricular block necessitates a biological solution to avoid the repetitive operations, which influence the quality of life of the children. Biomaterial science and cell delivery systems such as fibrin gel can be used as a state-of-the-art method for this aim. Fibrin is a plasma membrane protein that has been widely used because of its high biocompatibility [22,23]. In particular, fibrin gel has been widely used for in vitro tissue formation [24,25] for different applications which include wound reparation [26], drug delivery systems [27], cell delivery [28,29], gene delivery [30], differentiation in tissue engineering [31] and patterning [32]. However, fibrin composites lack in terms of mechanical properties compared to other polymers offering equal biocompatibility and biodegradability [33,34].

In this study, we are describing the method optimization of embedding induced pluripotent stem cell cardiomyocytes (iPSC-CMs) together with smooth muscle cells and endothelial cells for the preparation of an autonomous cylindrical construct. This study aims to assess the influence of the cell composition, the diameter and the cyclic mechanical stimulation on the beating frequency of the BioPacer constructs and also on the expression of important cell markers indicating the construct with the best performance. For the preparation of the BioPacer constructs we used a micro-molding technology described previously by Keijdener et al. The cell density of each construct is described in Table 1.

Different diameters were used for the molding of the hydrogels in the silicon tubes and also different cell compositions of the BioPacer were prepared for the assessment of the diameter and the stretch to the beating frequency of the constructs. Additionally, 2 types of constructs were subject to cyclic mechanical stretch and were also tested for the expression of iPSC-CM, HUVSMC and HUVEC markers.

## 2. Results and Discussion

### 2.1. Cell Infiltration and Proliferation

**Figure 1:**
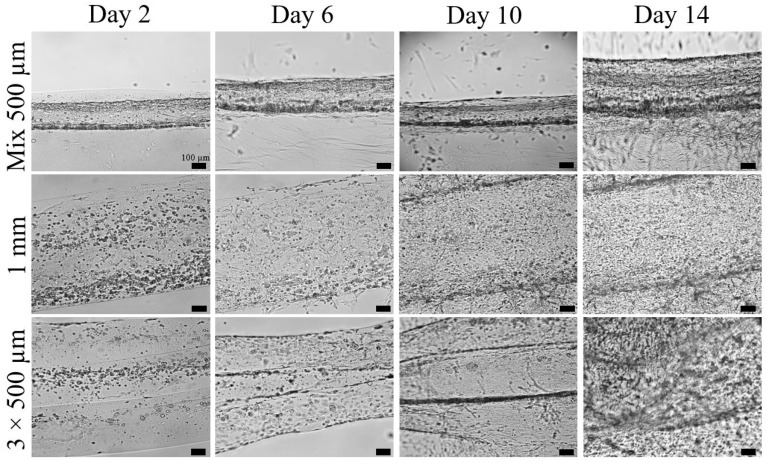
Bright-field images of three different mix configurations of the BioPacer for days 2, 6, 10 and 14 of the cultivation period. First row: Mix 500 µm sample. Second row: 1 mm sample. Third row: 3 × 500 µm sample.

For the investigation of the cell infiltration in the fibrin gel, three different BioPacer mix types of samples were prepared. The 1 mm (G7) and the Mix 500 µm (G2) samples had the same initial diameter on day 2 of cultivation. Even if the 3 × 500 µm (G6) sample had a bigger initial diameter, on day 6 of cultivation a contraction of the fibrin matrix was observed and that is why the diameter was decreased (Figure 1). This trend appeals to all the 3 types of samples. On the 10th day of cultivation, the cells and particularly the smooth muscle cells were expanded out of the cylindrical structure and they started attaching and growing on the surface of the well plate. On day 14 of cultivation and mostly for the G7 samples and for the G6 which contain the initial higher number of HUVSMCs, the proliferation was also higher and the HUVSMCs created multiple layers around the fibrin gels (right column of Figure 1). As a consequence, the percentage of cell integration was effective for the different diameters of the BioPacer sample and the samples could be functional in terms of integration in the fibrin gel for various configurations.

### 2.2. iPSC-CM Markers for the BioPacer

**Figure 2:**
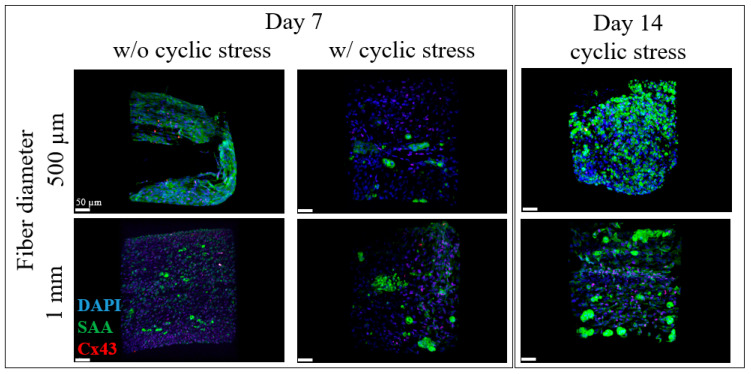
DAPI (blue), SAA (green) and Cx43 (red) for the BioPacer samples without and with cyclic stress cultivated for 7 days and with cyclic stress for 14 days.

One of the first steps for the effect of stretching of the iPSC-CMs was the investigation of the effect of stretching for different cultivation periods on the samples. In Figure 2 the BioPacer sample 500 µm and the 1 mm BioPacer are represented without and with cyclic stress cultivated for 7 days and with cyclic stress for 14 days. Cross-sectional cuts are presented in Supplementary Figure S1. 

The 500 µm sample showed that the expression of SAA from the iPSC-CMs was relatively high comparing the 500 µm BioPacer sample with applied stretch. However, the expression of Cx43 in the 1 mm stretch sample was higher in the 1 mm sample without applied stretch, indicating that the stretching of the samples positively affects the electrical coupling among the iPSC-CMs. Another important factor was the longer cultivation of the samples. Moreover, the influence of stretching was much more visible in the 1 mm BioPacer as the nuclei were oriented to the direction of the stretch. Moreover, the SAA was not equally expressed by all the cells embedded in the fibrin gel. Comparing the 500 µm sample, the SAA production was distributed in a more regular pattern, as expected from the absence of stretch. At the same time, the Cx43 signal was higher for the samples cultivated for 14 days, as the iPSC-CMs needed some days to regulate their metabolism and their normal function. 

**Figure 3:**
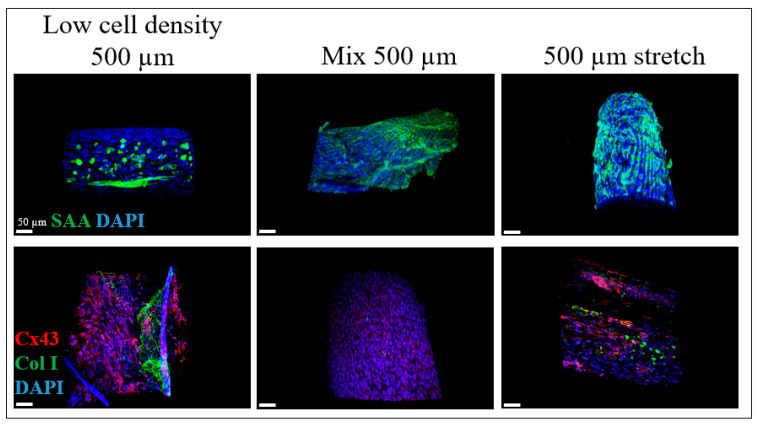
DAPI (blue), SAA (green), Cx43 (red) and collagen I (green) staining. Left: Low cell density 500 µm (G1), middle: Mix 500 µm (G2), right: 500 µm stretch (G4).

As a further step, we investigated the expression of the SAA, Cx43 and col I according to the concentration change of the iPSC-CMs in the constructs, the composition and the applied cyclic stretch. In Figure 3 (first row), the respective configurations are shown for the SAA expression of the Low cell density 500 µm (G1), the Mix 500 µm (G2) and the 500 µm stretch sample (G4). Cross-sectional cuts are presented in Supplementary Figure S2. 

The SAA expression of the G1 sample was concentrated on one side of the fiber, where the iPSC-CMs, which are responsible for the SAA-specific signal, are also located. In these fibers, even if the concentration of the iPSC-CMs is half of the concentration in the G2 sample, the SAA signal was still significant. In Figure 3, there was an overall distribution of the SAA signal reflecting the difference in the molding process by mixing all the cell types in a common matrix. In the samples where the number of the iPSC-CMs was low, the collagen expression from the smooth muscle cells was more profound as there was more space for the disposal of fibroblasts in the diameter of the cylindrical structure, unlike the conventional BioPacer samples, where the collagen amount was a bit mitigated from the presence of a higher number of iPSC-CMs which were present in the BioPacer samples. At the same time, the Cx43 signal was stronger in the G1 than in the G2 sample, a fact that is reflecting the effect of fibroblast proliferation in the G2 sample. Furthermore, as for the effect of the applied cyclic stretch, the expression of collagen was the strongest among all the other samples, as it constitutes the direct consequence of the applied stretch. Simultaneously, the Cx43 signal was still present, but not as in a high amount as in the G1 sample.

**Figure 4:**
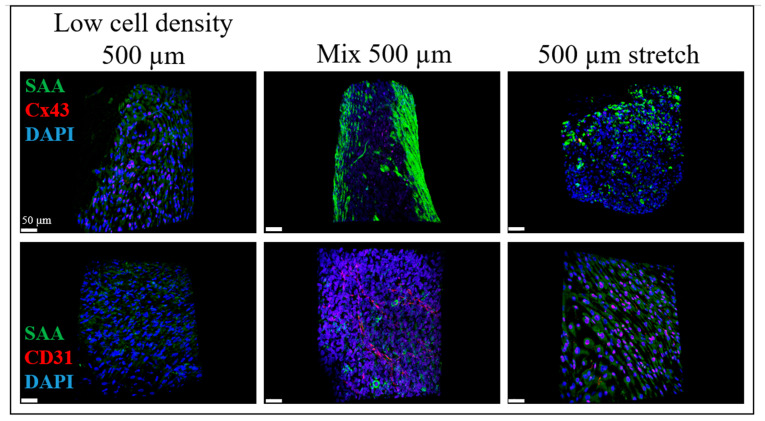
DAPI (blue), SAA (green), Cx43 (red) and CD31 (red) staining. Left: Low cell density 500 µm (G1), middle: Mix 500 µm (G2), right: 500 µm stretch (G4).

Additionally, the SAA and Cx43 markers for the iPSC-CMs appeared to be in a good correlation with the CD31 marker for the endothelial cells that were present in the cylindrical structures of the BioPacer. In Figure 4, the SAA signal produced by the iPSC-CMs was stronger than the Cx43 signal for the 3 types of samples analyzed. Cross-sectional cuts are presented in Supplementary Figure S3. At the same time, the Cx43 seemed to be stronger in the G4 sample, as the iPSC-CMs gathered in the middle of the cylindrical core. Furthermore, the SAA expression in the G4 sample appeared to be stronger than in the G1 and G2 samples. Moreover, the SAA spots were well oriented in the G4 sample, unlike the G1 and the G2 samples. Comparing the G1 and the G2 samples, the CD31 cobblestone structure was clear in the G2 sample, unlike the G1 sample where the CD31 expression was too low. Additionally, the CD31 expression pattern followed the direction of the stretch in the G4 sample. 

In order to better assess the qualitative results from the two photon images of the samples, volume quantification was performed using the most important markers expressed in the cells used in the present study (HUVSMCs, HUVECs and iPSC-CMs) such as SAA, Cx43, Col, CD31 and DAPI. 

Particularly, the applied stretch consists of an influencing factor for the percentage of DAPI, as there is a significant difference between the 1 mm (G7) and 1 mm stretch (G8) samples. Moreover, the Cx43 signal did not show significant differences. On the other hand, the expression of SAA had the highest value for the G8 sample and was significantly different from the respective value of the G7 sample. This could be explained by the fact that the iPSC-CMs the G7 could not produce the same amount of SAA as the G8 sample, because of the inhibitory proliferation of the HUVSCMs and the expansion of the latest ones outside the fibrin matrix. The highest proliferation rate of the HUVSMCs and the lack of proliferation of the iPSC-CMs prohibits the production of SAA, an indicator of their maturity. However, the G8 sample represented a higher expression of SAA, as the mechanical stimulation of the samples could trigger them and catalyze the production of SAA from the side of the iPSC-CMs. 

**Figure 5:**
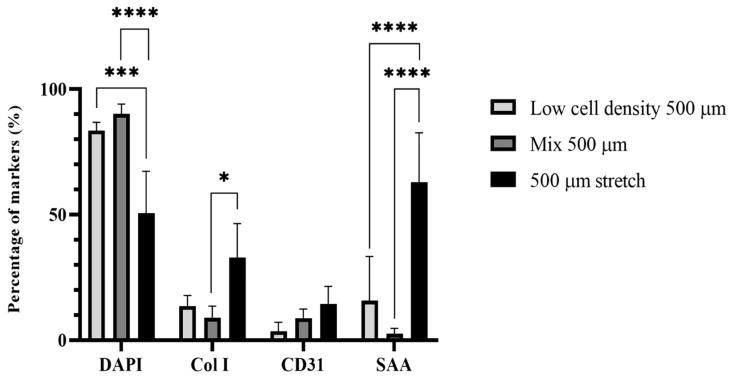
Quantification of DAPI, SAA, CD31 and Col I for the Low cell density 500 µm (G1), Mix 500 µm (G2), 500 µm stretch sample (G4) (Data are expressed as mean ± SD, *n* = 3, * *p* < 0.05,*** *p* < 0.001, **** *p* < 0.0001).

Furthermore, col I and CD31 were also quantified according to their volume in the samples to evaluate the function of HUVSMCs and HUVECs in the G1, G2 and G4 samples (Figure 5). As observed in Figure 5, the expression of the col I was the highest for the G4 sample, as the applied stretch promoted the higher expression of collagen from the HUVSMCs. This difference in the value was significantly different from the expression of the col I in the G2 sample, even if it contained the same amount of HUVSMCs as the G4 sample. This gives more importance to the fact that the applied stretch affected the expression of the col I. The lowest collagen expression was observed for the G2 sample. The reason is that the concentration of HUVSMCs in the G2 sample was lower than the G1 sample and the subsequent proliferation and production of col I from the HUVSMCs was also lower. At the same time, in the mixture of the cells in the cylindrical fibrin matrix, the geometric positioning of the HUVSMCs also affected the production of collagen as the distance between the single cells is longer and this does not facilitate the synergistic action for the production of extracellular matrix. 

Furthermore, the profile of the expression of SAA had the same tendency as the expression of col I. The SAA had the greatest value in the G4 sample, because of the mechanical stimulation of the BioPacer. The result was significantly different from the G1 sample, as the amount of cells is significantly different than the G4 sample. Similar was the behavior of the G2 sample, with a highly significant difference compared with the G4 sample. This is explained by the fact that the iPSC-CMs were located in the cylindrical structure more isolated than in the G1 sample. Consequently, they act more individually than they may do in the G1 and in the G4 sample, where the mechanical loading contributes to the matrix remodeling.

### 2.3. Beating Frequency

**Figure 6:**
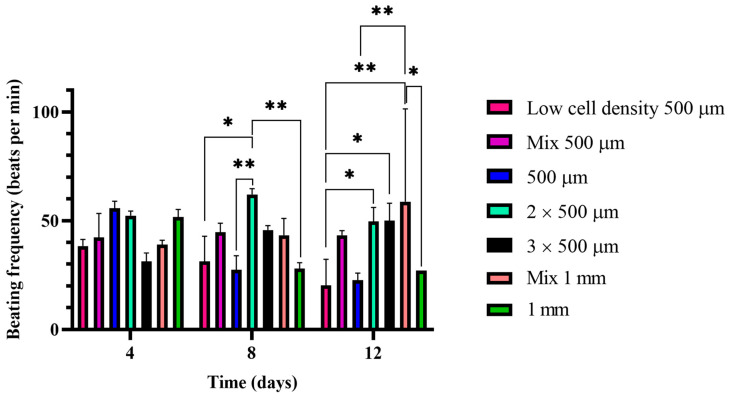
Beating frequencies for the BioPacer samples (Low cell density 500 µm (G1), Mix 500 µm (G2), 500 µm (G3), 2 × 500 µm (G5), 3 × 500 µm (G6), Mix 1 mm (G9) and 1 mm (G7) on the Day 4, 8 and 12 of cultivation (Data are expressed as mean ± SD, *n* = 3, * *p* < 0.05, ** *p* < 0.01.

During the cultivation period, we evaluated the beating frequency of the constructs. As observed during the cultivation period, the constructs started the synchronized beating after the second day of cultivation, reaching their maximum level after the first week of cultivation. The highest beating frequency was shown for the 2 × 500 µm sample (G5) and was observed on the 8th day of cultivation, while the lowest beating frequency value was shown for the G1 sample, where the concentration of the iPSC-CMs is half of the amount in the rest of 500 µm samples (Figure 6). The samples in which the beating frequency had a slightly increasing tendency were the G6 and the G2 sample. The beating frequency for the G5 sample had also the same increasing tendency, but the value was slightly decreased on the 12th day. On the contrary, for the G1, the G3 and the G7 samples the beating frequency had been decreasing during the cultivation period (Figure 6).

Looking more deeply at the beating frequency capacities of the different samples, until the 4th day, there were no significant changes among the samples, even if the samples started the homogeneous and synchronized beating until the 4th cultivation day. On the contrary, on the 8th day, there was a significant difference between the G1 sample and the G5 sample, as the latter contained a higher concentration of iPSC-CMs promoting the increase in the beating frequency over the cultivation period. At the same time, the G7 sample presented also higher significant difference than the G5 sample, as the higher proliferation of HUVSMCs prohibited the action of the high number of iPSC-CMs, by isolating their signal at a higher extent than needed. However, most of the changes appeared on the 12th day of the cultivation period. For instance, the G1 sample had a significantly different beating frequency than the G5 and G6 samples. The reason is that the higher concentration of iPSC-CMs stabilized the threshold of the beating frequency values of the G5 and G6 samples (Supplementary Video S1). Moreover, the beating frequency of the Mix 1 mm sample (G9) was significantly higher than the respective G7 value, indicating that the mixing of the samples acts as a promoting factor for the maintenance of the beating frequency. Additionally, there was a highly significant difference between the G1 and the G9 sample. The main reason appeared to be the increased iPSC-CM concentration in the samples in combination with the mixing during the molding. Simultaneously, the same is the behavior of the G3 sample, as in this case only one of the fibers that constitute the BioPacer was beating, while the other one acted as a mechanical support and signal isolator of the electrical signal. 

### 2.4. Glucose and Lactate Measurements

**Figure 7:**
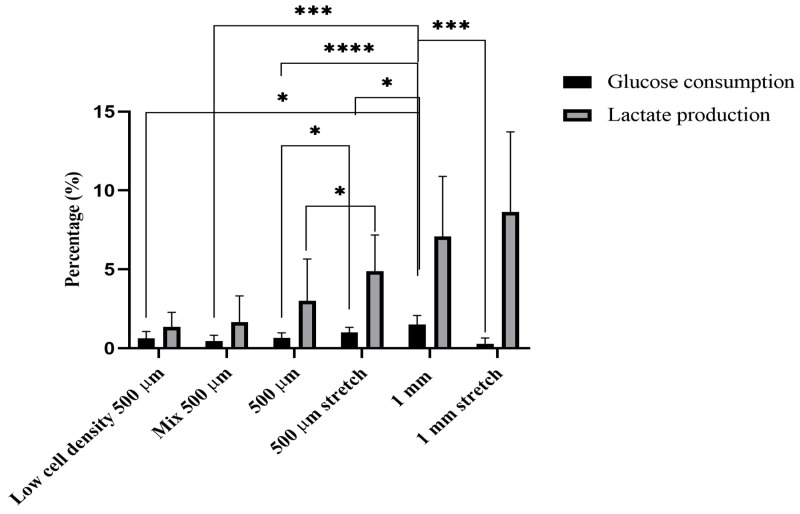
Glucose and lactate levels for the BioPacer constructs. Percentage of glucose consumption and lactate production for the Day 14 of the cultivation period. Data are expressed as mean ± SD, *n* = 3 (* *p* < 0.05, *** *p* < 0.001, **** *p* < 0.0001).

The glucose and lactate levels were measured for the different configurations of the BioPacer for the constructs cultivated for 14 days. In Figure 7, the consumption of glucose is plotted for the six different configurations of the BioPacer. The highest level of glucose consumption was achieved for the G7 sample presenting significant differences among all the other sample types. For the samples with different diameters, there are more significant differences observed between the samples (G1 and G7, G2 and G7, G3 and G7, G4 and G7) than the differences when comparing samples with the same diameter (G3 and G4, G7 and G8). As a result, the diameter plays an important role in the glucose consumption of the samples. Furthermore, the lactate production value of the G4 sample was reported higher than the G3 sample, reflecting the positive effect of the applied stress in the metabolism of the small diameter samples. The cell concentration in the G1 samples is the half value of the concentration in the G3 samples and one-fourth less than the value of the cell concentration in the cell concentration in the G7 samples. Therefore, the glucose provided by the new media change was a lot more than the cells embedded in the G1 samples needed to consume. Concerning the G4 sample there was higher glucose consumption and simultaneously higher lactate production than the G3 sample and both metabolites followed the same trend with slight differences in their values during the cultivation period. However, a highly significant difference in glucose consumption was shown between the G7 and the G8 samples. In the G7 samples, the glucose levels were decreasing while the lactate consumption was increasing during the first week of the cultivation. This can be explained by the fact that the iPSC-CMs were isolated by the high increase in the HUVSMCs in the constructs after the first week of cultivation. Therefore, the proliferation of HUVSMCs acted as an inhibitory factor to the effective iPSC-CM function, as the last ones seemed to affect glucose consumption and lactate production to a high extent. 

### 2.5. Discussion

In this study, we presented the expression of the iPSC-CM markers for the ex-vivo cultivation of fibrin hydrogels embedded with iPSC-CMs, HUVECs and HUVSMCs. There are different configurations of the BioPacer sample that were tested for glucose and lactate measurements to define the viability of the construct and for cell morphology to visualize the cell expansion inside the fibrin matrix. Additionally, the expression of iPSC-CM markers SAA, Cx43, col I and CD31 were investigated for the HUVSMCs and HUVECs, respectively. Moreover, the beating frequency of the iPSC-CMs in the fibrin hydrogels was evaluated. All these factors affect the performance of the BioPacer as a beating construct in vitro. 

Furthermore, the selection of the stretch sample confirmed that the stretch constitutes an important factor in the preparation of the BioPacer constructs. Uniaxial stretch has been proven to positively affect the cytosolic calcium levels in iPSC-CMs [35] increase the sarcomere length of iPSC-CMs [36] and successfully differentiated in vitro using mechanical stretch [37]. In our two photon microscope pictures, we assume that the expression of SAA, Cx43, CD31 and col I were respective to the cell concentration of the samples, the molding technique and also the stretch. Particularly, the uniaxial cyclic stretch affects the cell elongation of the cells along the fibrin gel tube in comparison to the static culture (Figure 2). Moreover, the DAPI, SAA, Cx43, Col I and CD31 expression in Figure 3 and Figure 4 were proportional to the low cell concentration of the iPSC-CMs, to the molding technique and to the applied uniaxial cyclic stretch. Our results for the stretcher were in accordance with another study where cardiac patches made of matrigel were subjected to cyclic mechanical stimulation. In this study, the expression of SAA and Cx43 is totally affected by the applied stretch on the cardiac patches and is definitely affecting the cell alignment [38]. On the other hand, another type of stretch, the cyclic sinusoidal strain increases their sarcomere orientation perpendicular to the axis of strain [39].

As a further step, we believe that the combination of an electrical stimulation system together with the mechanical stimulation achieved from our cyclic stretch bioreactor would serve as a complete construct with both types of stimulation achieving the ideal functionality of the cylindrical hydrogel. Recently, Dou et al. created a biosensing platform for the electrical stimulation of the iPSC-CMs with the simultaneous contraction and electrophysiology measurements of the monolayers [40]. Therefore, the monolayers corresponded well to the electrical stimulation, as the Cx43 signal for the electrical coupling and the SAA signal, which indicates the maturity of the cells, were expressed accordingly to the applied electrical stimuli. In another study, electrical and mechanical stimulation were also combined for translational applications [41].

Concerning the beating frequency of the samples, the G2 sample together with the G5 sample and the G6 sample performed more efficiently. As it was recently investigated, the presence of fibroblasts in the culture is beneficial for the electrical pacing of the constructs [42]. In our case, we used the fibroblasts to stabilize the fibrin core via collagen production, which was additionally increased after the applied stretch (Figure 6). Consequently, the increased fibroblast concentration led to decreased beating frequency over the culture. Moreover, the beating frequency of our constructs could also increase in case of electrical stimulation as reported in a study where iPSC-CM spheroids were mechanically and uniaxial cyclically stimulated [43]. Another study used special electrodes for the electrical stimulation of iPSC-CMs with the further aim to synchronize the beating [44].

As for the glucose and lactate measurements, the diameter of the samples plays an important role in glucose consumption. This is reflected by the fact that the glucose consumption of the G7 sample was significantly higher than the G3 sample value. At the same time, the glucose consumption of the G3 sample was significantly lower than the respective value of the G4 sample, indicating that the cell metabolism has been increased because of the stretch. However, the glucose consumption in the G7 samples is significantly higher than the respective value of the G8 sample, demonstrating that the effect of stretch on the consumption of glucose also depends on the diameter of the constructs. Moreover, the simultaneous comparison of the lactate results represented a highly significant difference between the G3 and the G4 sample. Consequently, for lactate production, only the stretch is a determinant factor that affects the cell metabolism and not the diameter. In other studies investigating the interactions between smooth muscle cells and endothelial cells, the glucose and lactate levels varied during the culture [45]. Considering only the first week of the cultivation period, the glucose levels changed slightly and the same tendency was observed for the lactate levels as stated in a study of the biological vasculature using endothelial cells in a microfluidic device [46]. Particularly, our results for the different tendencies between glucose consumption and lactate production (Figure 7) over the entire culture period may be explained by the different initial cell concentrations in the constructs. For example, Heywood et al. showed that the glucose and lactate rates were affected by the cell concentration in particular chondrocytes [47]. Specifically in our study, the lactate levels were either slightly increasing (G4 sample) or abruptly increasing after the first week of the culture period. However, there is further investigation needed for these results, as it has been proven that the high levels of lactate in the culture are correlated with fibrin clotting in neutrophil extracellular trap formation [48]. Last but not least, considering the cultivation time, Zheng et al. proved that the glucose and lactate levels increased during the first minutes of the cultivation in a fibrin gel acting as a barrier for tumor cell migration based on the property of fibrin to clot [49]. Furthermore, our glucose and lactate results for the G4 sample during the culture period followed a similar tendency to a study where fibrin gels in the geometry of the heart valve were dynamically cultivated and the rates of both glucose and lactate do not steeply change [50]. Particularly for the iPSC-CMs which were the main component of all the constructs, they have been reported to maintain the glucose levels high in the culture and the lactate levels relatively low during the first days of cultivation in a study for drug testing [51]. That is why we concluded that the iPSC-CMs cannot provide specific information for the explanation of these results when they are embedded in the fibrin gel.

## 3. Conclusions

We managed to fabricate cylindrical constructs which contain three cell types (HUVSMCs, HUVECs and iPSC-CMs) and subject them to cyclic stretch. The glucose and lactate levels of the constructs and the presence of iPSC-CM markers together with HUVEC and HUVSMC markers indicate the function of the constructs according to the diameter and the cyclic stretch. By adjusting the cell composition and concentration together with the cyclic stretch iPSC-CM embedded fibrin cylindrical hydrogels can be produced in vitro with the further aim to be used in the treatment of the atrioventricular block in pediatric patients.

## 4. Methods

### 4.1. Cylindrical Fibrin Gel Constructs

**Figure 8:**
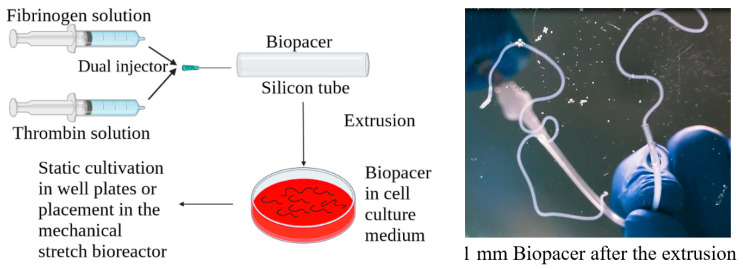
Conduction of the experiment.

500 µm and 1 mm diameter translucent silicone tubing was incubated overnight in 1% pluronic solution (Sigma-Aldrich, Saint Louis, MO, USA) at room temperature. The air was removed using a needle and a syringe (B. Braun, Melsungen, Germany). The concentration of the cell suspension was 3 million/mL, 1 million/mL and 15 million/mL for HUVSMCs, HUVECs and iPSC-CMs, respectively. Lyophilized fibrinogen from human plasma (Sigma-Aldrich, Saint Louis, MO, USA) was dissolved in purified water and dialyzed against Tris-buffered saline (TBS) using Spectra/Por 1 tubing (Spectrum) with a molecular weight cut-off of 6000–8000 Da. After sterile filtration, the fibrinogen concentration was determined by measuring the absorbance at 280 nm using an Infinite M200 spectrophotometer (Tecan Group Ltd., Männedorf, Switzerland). The cells were detached from the flasks and resuspended in a solution consisting of 50% Tris-buffered saline (TBS) solution, 25% 40 U/mL thrombin (Sigma-Aldrich, Saint Louis, MO, USA) and 25% 50 mM CaCl_2_. The polymerization took place via a dual injection head and mixer (Medmix Systems AG, Switzerland) connected to two syringes containing the fibrinogen and the cell suspension, respectively (Figure 8). Additionally, the syringes were held together with Duploject double syringe holder (Tisseel, Baxter, United Kingdom). After 5 min, the fibers were extruded from the tubes by adding DMEM and were collected in a DMEM bath in a Petri dish. The fibers were cut into different lengths and were placed either in 6 well plates for the static cultivation or in the cyclic stretch bioreactor for the dynamic cultivation condition. The samples were cultivated with a medium consisting of 25% DMEM, 25% EGM-2 and 50% Plyricyte medium supplemented with L-ascorbic acid-2-phosphate (1.0 mM; Sigma) and antibiotic medium (ABM, Pan Biotech, Aidenbach, Germany). The medium was changed on days 2, 4, 6, 8, 10 and 12 and the constructs were cultivated either for 7 or 14 days to study the appropriate functionality of the BioPacer constructs. The procedure described above was followed for the preparation of the BioPacer configurations listed in Table 1.

### 4.2. Cell Culturing

Human umbilical vein smooth muscle cells (HUVSMCs) and human umbilical vein endothelial cells (HUVECs) were handled as described previously [52]. Human umbilical cords were obtained after written consent at the University Hospital Aachen, Aachen, Germany, and were provided by the RWTH Aachen University Centralized Biomaterial Bank (cBMB) according to its regulations, following RWTH Aachen University, Medical Faculty Ethics

Committee approval (cBMB project number 323). The medium used was Dulbecco’s Modified Eagle’s Medium (DMEM, Thermofischer, Waltham, MA, USA) supplemented with 10% FCS for the HUVSMCs and EGM (PromoCell, Heidelberg, Germany) supplemented with 1% FCS, basic Fibroblast Growth Factor, Insulin-like Growth Factor, Vascular Endothelial Growth Factor 165, Ascorbic Acid, Heparin and Hydrocortisone for the HUVECs, respectively. Both cell types were cultured in flasks and trypsin (Thermo Fisher, Waltham, MA, USA) was used for cell dissociation. For the HUVECs flasks were coated with 2% gelatin (Sigma-Aldrich, Saint Louis, MO, USA) before culture. The cells were cultured at 37 °C, 5% CO_2_, the medium was changed every 3 days and they were up to passage 5.

Induced pluripotent stem cell cardiomyocytes (iPSC-CMs) (Ncardia, Leiden, The Netherlands) and were cultured in 10 μg/mL fibronectin (Sigma-Aldrich, Saint Louis, MO, USA). TrypLE Select Enzym (1×, Thermofischer, Waltham, MA, USA) was used for the trypsinization. Plyricyte medium (Ncardia, Leiden, The Netherlands) was used for the cultivation of the iPSC-CMs.

### 4.3. Bioreactor Set-Up

**Figure 9:**
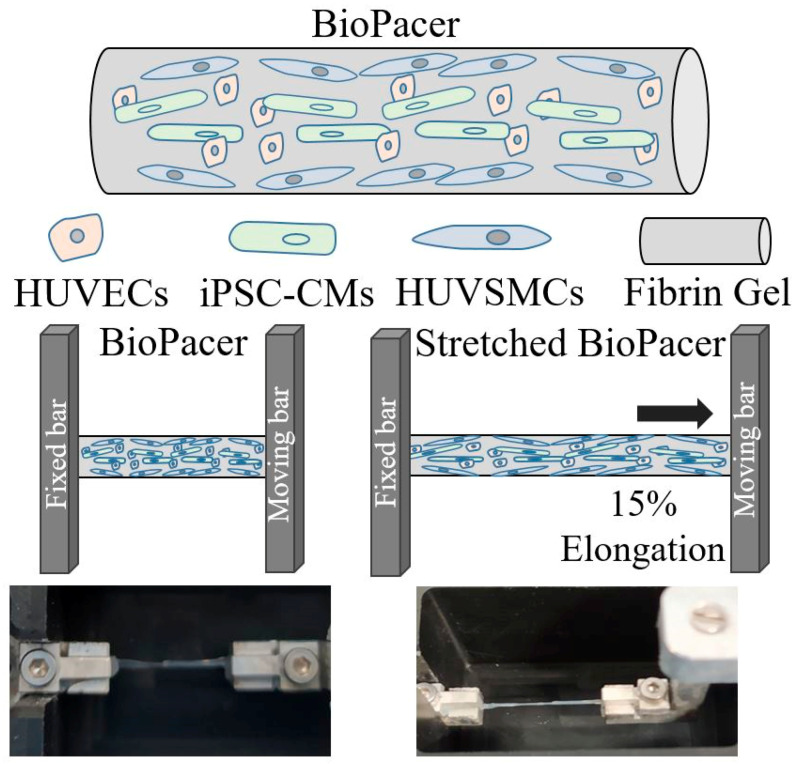
The BioPacer sample in the bioreactor system.

A housemade cyclic stretch bioreactor was constructed in the workshop of the Applied Medical Engineering Department of the Helmholtz Institute Aachen. The bioreactor consisted of a chamber with positions for 6 samples held by two edges using a metal holder and a tiny screw on one side. The bioreactor was sealed in a box whose walls were made of transparent PTFE for the less complicated observation of the experiment. The samples were stretched to 15% after 7 days of static cultivation. The setup is presented in Figure 9. The software and the electrical supply setup were provided by Igus (Cologne, Germany). The whole bioreactor system is presented in Supplementary Figure S4.

### 4.4. Glucose and Lactate Measurements

During the cultivation period, the glucose and lactate levels of the culture medium were measured using an Epoc Reader (Epoca, Ottawa, ON, Canada).

### 4.5. Immunohistochemistry

After cultivation, the samples were fixed in a 4% PFA solution (Carl–Roth, Karlsruhe, Germany) for 1 h and then washed 2 times with PBS (Thermofischer, Waltham, MA, USA). The cell membrane was permeabilized using 5% normal goat serum and 0.1% Triton X-100 (Sigma-Aldrich, Saint Louis, MO, USA) in PBS. The samples were incubated with the primary antibody and incubated overnight at 37 °C. Afterward, the samples were washed 2 times with PBS and incubated for 8 h at 37 °C. Furthermore, the samples were incubated with the secondary antibody following the same conditions applied for the primary antibody incubation. After the last washing step, the nuclei were stained with DAPI and incubated for 15 min at 37 °C and washed 3 times. The samples were stored at 4 °C in PBS containing ABM until visualization in a 1% antibiotic-antimycotic solution. In Table 2, the primary and secondary antibodies used are listed. Samples were embedded in 2% agarose gel in PBS and visualized by two-photon laser-scanning microscopy (TPLSM) using an Olympus FluoView 1000MPE with a 25× water objective (NA 1.05, Olympus, Tokyo, Japan), a mode-locked MaiTai DeepSee Titanium-Sapphire Laser (Spectra-Physics, Stahnsdorf, Germany) and FluoView FV 10 4.2 acquisition software.

### 4.6. Beating Frequency

Samples were placed in a Nikon Ti-Eclipse epifluorescence microscope TI-S-CON (Tokyo, Japan) at 37 °C and 5% CO_2_ using an Okolab heating box (Ottaviano, NA, Italy). A video with a duration of 1 min was recorded for each sample and the beating frequency per minute was calculated. The iPSC-CM spots beating simultaneously were selected for these measurements.

### 4.7. Quantification of the Markers Content

The Imaris software was used to evaluate the images captured at the two-photon laser-scanning microscope. The pixels were counted using the masking option in the surface’s menu for the three different colors (blue, red, green) and the whole set of volume and surfaces was used for the quantification of the different markers. The consumption of glucose and the production of lactate values were expressed as percentages based on the difference between the initial and the final value of every marker, respectively.

### 4.8. Statistics

All data were represented as mean-SD from three experiments. For the glucose and lactate measurements, uncorrected Fisher’s LSD test with one-way analysis of variance (ANOVA) was performed. For the quantification of the TPLSM results and the beating frequency measurements, Tukey’s multiple comparisons test with two-way analysis of variance (ANOVA) was selected. A *p*-value < 0.05 was considered statistically significant.

## Figures and Tables

**Table 1:** BioPacer samples.

Sample Type	Fiber Number	[iPSC-CMs] (Cells/Fiber)	[HUVSMCs] (Cells/Fiber)	[HUVECs] (Cells/Fiber)
Low cell density 500 µm (G1)	2	150,000	60,000	20,000
Mix 500 µm (G2)	1	300,000	60,000	20,000
500 µm (G3)	2	300,000	60,000	20,000
500 µm stretch (G4)	2	300,000	60,000	20,000
2 × 500 µm (G5)	2	300,000		
3 × 500 µm (G6)	3	300,000		
1 mm (G7)	2	500,000	120,000	60,000
1 mm stretch (G8)	2	500,000	120,000	60,000
Mix 1 mm (G9)	1	500,000	120,000	60,000

**Table 2:** Antibodies for the staining of the samples.

Antibody	Dilution	Supply
Anti-α-Actinin (sarcomeric), mouse monoclonal	1:50	Sigma-Aldrich
Connexin 43 (polyclonal)	1:50	Thermofischer
Connexin 43 Monoclonal Antibody (3D8A5)	1:50	Thermofischer
Anti-CD31 (PECAM-1), mouse monoclonal	1:100	Sigma-Aldrich
CD31 rabbit polyclonal	1:100	Abbiotec
α-smooth muscle actin (monoclonal, clone 1A4)	1:1000	Sigma-Aldrich
Anti-Collagen I antibody (ab34710)	1:200	Abcam

## Data Availability

Data associated with this study is available upon request to the corresponding authors.

## Supplementary Materials

The following supporting information can be downloaded at: https://www.mdpi.com/article/10.3390/gels9090677/s1, Figure S1: SAA (red), Cx43 (green) and DAPI (blue) for the Biopacer samples without and with cyclic stress (cross-sectional cuts), Figure S2: DAPI (blue), SAA (green), Cx43 (red) and collagen I (green) staining. Left: Low cell density 500 µm, middle: Mix 500 µm, right: 500 µm stretch (cross-sectional cuts), Figure S3: DAPI (blue), SAA (green), Cx43 (red) and CD31 (red) staining. Left: Low cell density 500 µm, middle: Mix 500 µm, right: 500 µm stretch (cross-sectional cuts), Figure S4. Bioreactor set-up, Video S1: G6 (3 sample at Day 8).
