# Supplementary material for: Influence of Diameter and Cyclic Mechanical Stimulation on the Beating Frequency of Myocardial Cell-Laden Fibers
Source: Gels. 2023 Aug 23;9(9):677. doi: 10.3390/gels9090677 (PMC10528042; doi:10.3390/gels9090677)
Supplement: Supplementary file 1 [file gels-09-00677-s001.zip › gels-2545323-supplementary.pdf]

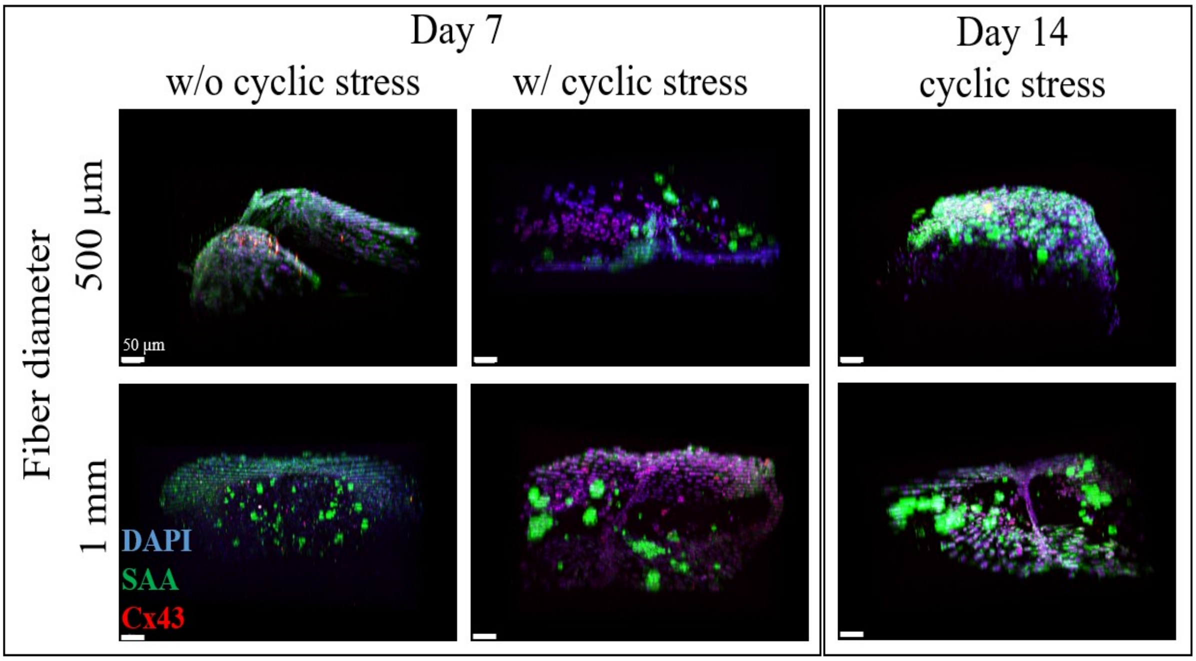


Figure S1: SAA (red), Cx43 (green) and DAPI (blue) for the Biopacer samples without and with cyclic stress (cross-sectional cuts)

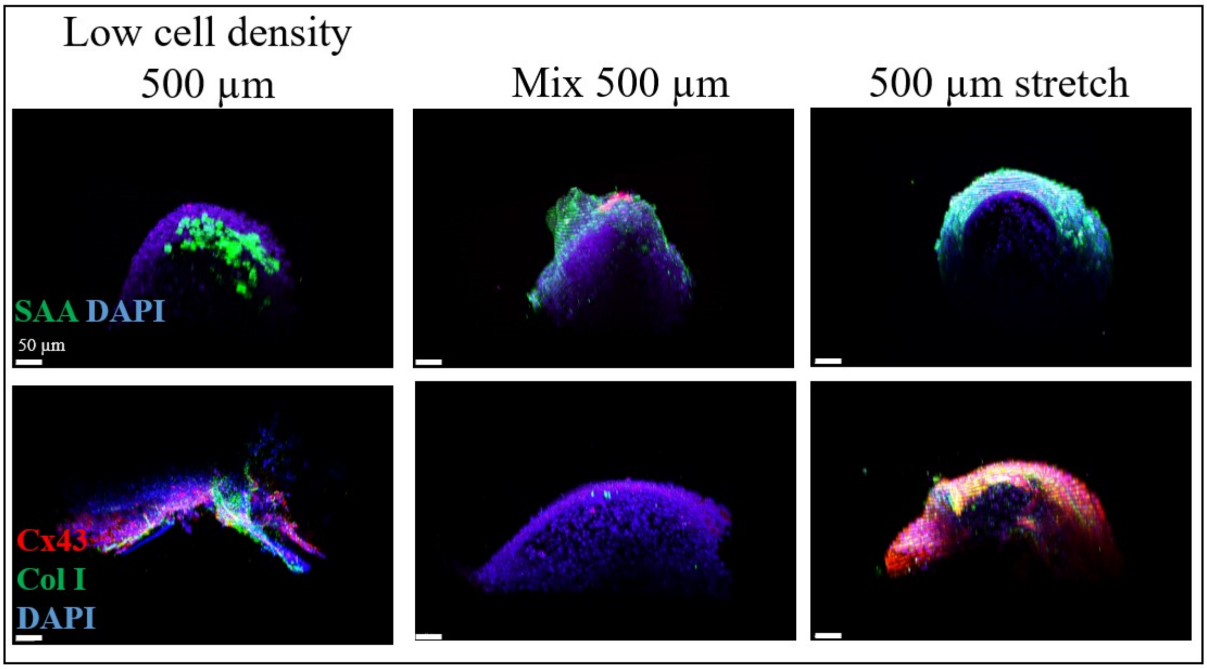


Figure S2: DAPI (blue), SAA (green), Cx43 (red) and collagen I (green) staining. Left: Low cell density 500 µm, middle: Mix 500 µm, right: 500 µm stretch (cross-sectional cuts).

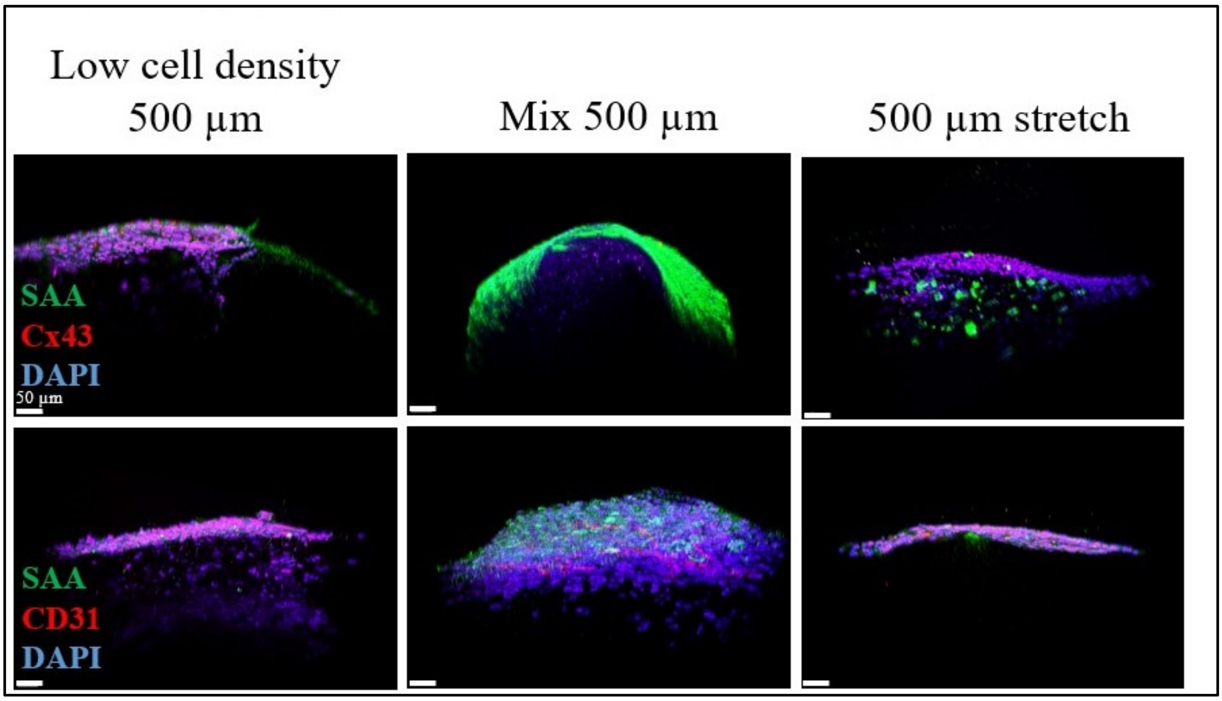

Figure S3: DAPI (blue), SAA (green), Cx43 (red) and CD31 (red) staining. Left: Low cell density 500 µm, middle: Mix 500 µm, right: 500 µm stretch (cross-sectional cuts)

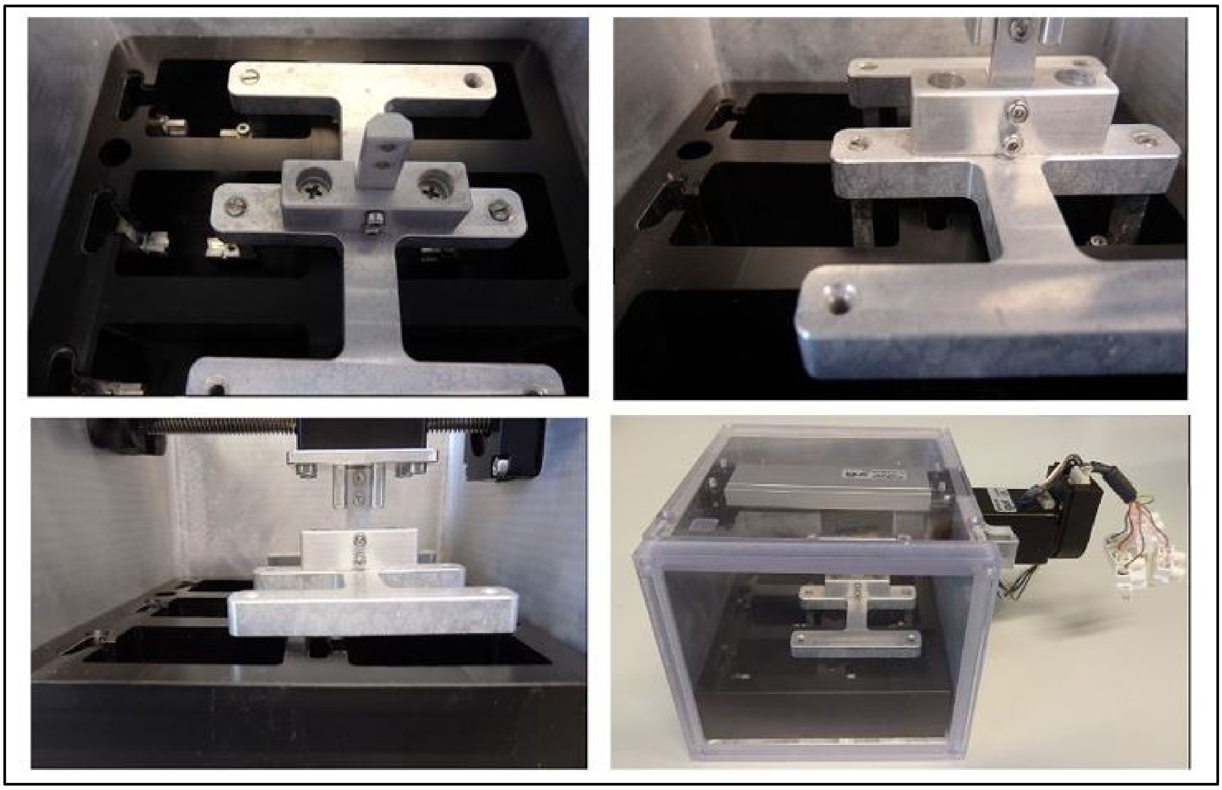
Figure S4: Bioreactor set-up
